# Clinicopathological predictors of recurrence in nodular and superficial spreading cutaneous melanoma: a multivariate analysis of 214 cases

**DOI:** 10.1186/s12967-017-1332-3

**Published:** 2017-11-07

**Authors:** Maria A. Pizzichetta, Daniela Massi, Mario Mandalà, Paola Queirolo, Ignazio Stanganelli, Vincenzo De Giorgi, Giovanni Ghigliotti, Stefano Cavicchini, Pietro Quaglino, Maria T. Corradin, Pietro Rubegni, Mauro Alaibac, Stefano Astorino, Fabrizio Ayala, Serena Magi, Laura Mazzoni, Maria Ausilia Manganoni, Renato Talamini, Diego Serraino, Giuseppe Palmieri, on behalf of the Italian Melanoma Intergroup (IMI)

**Affiliations:** 1 Division of Oncology B, CRO Aviano National Cancer Institute, Via Franco Gallini 2, 33081 Aviano, Italy; 2 Division of Pathological Anatomy, Department of Surgery and Translational Medicine, University of Florence, Florence, Italy; 3 Unit of Medical Oncology, Papa Giovanni XXIII Hospital, Bergamo, Italy; 4 Department of Medical Oncology, National Institute for Cancer Research, IRCCS San Martino, Genoa, Italy; 5 Skin Cancer Unit, Istituto Tumori Romagna (IRST), Meldola, Italy; 6 Department of Dermatology, University of Florence, Florence, Italy; 7 Clinic of Dermatology, IRCCS San Martino-IST, Genoa, Italy; 8 Department of Dermatology, Fondazione Ospedale Maggiore Policlinico IRCCS, Milan, Italy; 9 Dermatologic Clinic, Dept Medical Sciences, University of Torino, Turin, Italy; 10 Division of Dermatology, Pordenone Hospital, Pordenone, Italy; 11 Department of Dermatology, University of Siena, Siena, Italy; 12 Department of Dermatology, University of Padova, Padua, Italy; 13 Division of Dermatology, Celio Hospital, Rome, Italy; 14 National Cancer Institute, “Fondazione G. Pascale”-IRCCS, Naples, Italy; 15 Department of Dermatology, ASST degli Spedali Civili di Brescia, Brescia, Italy; 16 Unit of Epidemiology and Biostatistics, CRO Aviano National Cancer Institute, Aviano, Italy; 17 Unit of Cancer Genetics, Institute of Biomolecular Chemistry (ICB), National Research Council (CNR), Sassari, Italy; 18 Department of Dermatology, University of Parma, Parma, Italy

**Keywords:** Nodular melanoma, Superficial spreading melanoma, Prognostic indicators, Recurrence

## Abstract

**Background:**

Nodular melanoma (NM) accounts for most thick melanomas and because of their frequent association with ulceration, fast growth rate and high mitotic rate, contribute substantially to melanoma-related mortality. In a multicentric series of 214 primary melanomas including 96 NM and 118 superficial spreading melanoma (SSM), histopathological features were examined with the aim to identify clinicopathological predictors of recurrence.

**Methods:**

All consecutive cases of histopathologically diagnosed primary invasive SSM and NM during the period 2005–2010, were retrieved from the 12 participating Italian Melanoma Intergroup (IMI) centers. Each center provided clinico-pathological data such as gender, age at diagnosis, anatomical site, histopathological conventional parameters, date of excision and first melanoma recurrence.

**Results:**

Results showed that NM subtype was significantly associated with Breslow thickness (BT) at multivariate analysis: [BT 1.01–2 mm (OR 7.22; 95% CI 2.73–19.05), BT 2.01–4 mm (OR 7.04; 95% CI 2.54–19.56), and BT > 4 mm (OR 51.78; 95% CI 5.65–474.86) (p < 0.0001)]. Furthermore, mitotic rate (MR) was significantly correlated with NM histotype: [(MR 3–5 mitoses/mm^2^ (OR 2.62; 95% CI 1.01–6.83) and MR > 5 mitoses/mm^2^ (OR 4.87; 95% CI 1.77–13.40) (p = 0.002)]. The risk of recurrence was not significantly associated with NM histotype while BT [BT 1.01–2.00 mm (HR 1.55; 95% CI 0.51–4.71), BT 2.01–4.00 mm (HR 2.42; 95% CI 0.89–6.54), BT > 4.00 mm. (HR 3.13; 95% CI 0.95–10.28) (p = 0.05)], mitotic rate [MR > 2 mitoses/mm^2^ (HR 2.34; 95% CI, 1.11–4.97) (p = 0.03)] and the positivity of lymph node sentinel biopsy (SNLB) (HR 2.60; 95% CI 1.19–5.68) (p = 0.007) were significantly associated with an increased risk of recurrence at multivariate analysis.

**Conclusions:**

We found that NM subtype was significantly associated with higher BT and MR but it was not a prognostic factor since it did not significantly correlate with melanoma recurrence rate. Conversely, increased BT and MR as well as SNLB positivity were significantly associated with a higher risk of melanoma recurrence.

## Background

The incidence of melanoma is increasing worldwide with highest rates in northern Europe, United States and Australia [1]. The most commonly prognostic parameters in the recent AJCC/UICC staging model are breslow thickness (BT), ulceration and microscopic satellite while mitotic rate (MR) is an additional attribute that may be used in decision-making [2]. Nodular melanoma (NM) presenting with vertical growth phase without evidence of an initial radial growth phase, grows faster than melanomas associated with a radial growth phase such as superficial spreading melanoma (SSM) and lentigo malignant melanoma (LMM) [3].

According to the new taxonomy framework, NM may occur in sun-exposed skin without or with low cumulative sun-induced damage (low-CSD) or develop on skin with cumulative sun-induced damage (high-CSD) [4]. NM account for most thick melanomas and because of their frequent association with ulceration, fast growth and high mitotic rate, substantially contribute to melanoma-related mortality [3, 5–7].

LMM and SSM have been found to have a better prognosis than NM, however, when comparing for melanoma thickness, a significant difference between the subtypes was not found [8]. SSM and NM are believed to represent sequential phases of linear progression from radial to vertical growth. Clinical, pathological and epidemiologic evidences suggest, however, that SSM and NM might be the result of independent pathways of tumor development and underlying molecular differences between the two subtypes that may also contribute to the disparate outcomes.

The aims of the current study were to evaluate clinical and histopathological features associated with NM and SSM and to identify independent clinicopathological prognostic factors in a multicentre series of 214 primary melanomas including 96 NM and 118 SSM.

## Methods

The study series included only a limited fraction of the consecutive cases of histopathologically diagnosed primary invasive SSM and NM, presented with the combination of clinical and dermoscopic images and complete clinical history, observed during the period 2005–2010, in 12 Italian Melanoma Intergroup (IMI) centers. In order to guarantee sample homogeneity, only SSM and NM cases in patients with at least 5 years of follow-up, by the end of 2015 were included into the study. Each center provided clinical and pathological data such as gender, age at diagnosis, melanoma site, date of excision, pathological conventional parameters and date of first melanoma recurrence. By the beginning of January 2016, all information from the 12 centers was merged into a database at the Cancer Epidemiology Unit (R.T.) of the Centro di Riferimento Oncologico, Aviano (Italy), with a new identification link to the patient information on clinical features and histopathological diagnosis.

### Statistical analyses

Statistical analysis was performed by means of SAS statistical software 9.1 (SAS Institute Inc., Cary, NC, USA). Odds ratios (ORs) and corresponding 95% confidence intervals (CIs) were computed by unconditioned logistic regression to evaluate differences in the distribution of histopathological features of NM vs SSM. The significance of OR (β parameter) was tested through Wald Chi square. Statistically significant variables in the univariate analysis were included in the multivariate model. Tests for trend were based on the likelihood-ratio test between the models with and without a linear term for each variable of interest. The probability of recurrence was computed from the date of melanoma diagnosis to the date of first relapse or last follow-up. The curves of probability of recurrence were conducted by the Kaplan–Meier method and the differences were assessed with the log-rank test [9]. In addition, the differences were also tested in univariate and multivariate analyses using the Cox proportional hazards model to compute the hazard ratio (HR) and corresponding 95% confidence interval (CI) [10]. The significance of HR (β parameter) was tested through Wald Chi square. All results were considered statistically significant for values of p ≤ 0.05 (two-tailed test).

## Results

Of 214 primary cutaneous melanoma, 96 were NM and 118 were SSM. The study included 214 patients (118 men, 96 women) with a median age of 61 years (range: 21–96 years) for patients with NM and 57 years (range: 16–92) for patients with SSM. The sites of primary melanoma included head and neck (n = 17); limb (n = 84); and trunk (n = 113). We observed no differences between NM and SSM with regard to the distribution by sex, age, sites of melanomas, previous personal or family history of melanoma (data not shown).

Table 1 shows the univariate and multivariate analysis (OR) of main histopathological features of NM vs SSM. Multivariate analysis showed that BT was significantly associated with NM subtype: [BT 1.01–2 mm (OR 7.22; 95% CI 2.73–19.05), BT 2.01–4 mm (OR 7.04; 95% CI 2.54–19.56), and BT > 4 mm (OR 51.78; 95% CI 5.65–474.86) (p < 0.0001)] (Table 1). Furthermore mitotic rate (MR) was significantly correlated with NM histotype: [(MR 3–5 mitoses/mm^2^ (OR 2.62; 95% CI 1.01–6.83) and MR > 5 mitoses/mm^2^ (OR 4.87; 95% CI 1.77–13.40) (p = 0.002)] (Table 1).

**Table 1:** Univariate and multivariate analysis (OR) of some histopathological features of nodular melanoma (NM) versus superficial spreading melanomas (SSMs)

	NM (N. 96)	SSMs (N. 118)	Univariate OR (95% CI)^a^	Multivariate^c^ OR (95% CI)^a^
Thickness (mm)				
≤ 1.00	9 (9.4)	72 (61.0)	1^b^	1^b^
1.01–2.00	24 (25.0)	22 (18.6)	8.73 (3.54–21.52)	7.22 (2.73–19.05)
2.01–4.00	44 (45.8)	22 (16.6)	16.00 (6.76–37.87)	7.04 (2.54–19.56)
> 4.00	19 (19.8)	2 (1.7)	76.00 (15.14–381.51)	51.78 (5.65–474.86)
χ^2^_1_ trend: p value			p < 0.0001	p < 0.0001
Ulceration				
Absent	46 (47.9)	96 (81.4)	1^b^	1^b^
Present	43 (44.8)	18 (15.2)	4.98 (2.59–9.57)	1.56 (0.67–3.62)
Unknown	7 (7.3)	4 (3.4)		
χ^2^_1_: p value			p < 0.0001	p = 0.3066
Regression				
Absent	65 (67.7)	67 (56.8)	1^b^	1^b^
Present	24 (25.0)	46 (39.0)	0.54 (0.30–0.98)	0.55 (0.25–1.22)
Unknown	7 (7.3)	5 (4.2)		
χ^2^_1_: p value			p = 0.0428	p = 0.1393
Mitoses (n/mm^2^)				
≤ 2	31 (32.3)	86 (72.9)	1^b^	1^b^
3–5	24 (25.0)	14 (11.9)	4.76 (2.19–10.34)	2.62 (1.01–6.83)
> 5	35 (36.5)	11 (9.3)	8.83 (4.00–19.49)	4.87 (1.77–13.40)
Unknown	6 (6.2)	7 (5.9)		
χ^2^_1_ trend: p value			p < 0.0001	p = 0.0015
TIL^d^				
Absent	29 (30.2)	34 (28.8)	1^b^	
Brisk	14 (14.6)	27 (22.9)	0.61 (0.27–1.37)	
Non-Brisk	46 (47.9)	48 (40.7)	1.12 (0.59–2.13)	
Unknown	7 (7.3)	9 (7.6)		
χ^2^_1_ trend: p value			p = 0.6174	

^a^Odds Ratio and 95% confidence interval (CI)

^b^Reference category

^c^Model including all significant terms in the univariate analysis

^d^Tumor infiltrating lymphocytes

Table 2 shows the univariate and multivariate analysis of recurrence (HR) of some histopathological features in patients with NM and SSM. Multivariate analysis showed that BT [BT 1.01–2.00 mm (HR 1.55; 95% CI 0.51–4.71), BT 2.01–4.00 mm (HR 2.42; 95% CI, 0.89–6.54), BT > 4.00 mm. (HR 3.13; 95% CI 0.95–10.28) (p = 0.05)], MR > 2 mitoses/mm^2^ (HR 2.34; 95% CI 1.11–4.97) (p = 0.03) and the positivity of sentinel lymph node biopsy (SNLB) (HR 2.60; 95% CI 1.19–5.68) (p = 0.007) were significantly associated with an increased risk of recurrence (Table 2).

**Table 2:** Univariate and multivariate analysis of recurrence (HR) of some histopathological features of patients with nodular melanoma (NM) and superficial spreading melanomas (SSMs)

	Recurrence		Univariate	Multivariate^c^
	Yes (N. 53)	No (N. 161)	HR (95% CI)^a^	HR (95% CI)^a^
Diagnosis				
SSMs	14 (26.4)	104 (64.6)	1^b^	1^b^
NM	39 (73.6)	57 (35.4)	4.09 (2.22–7.53)	1.62 (0.77–3.39)
χ^2^_1_: p value			p < 0.0001	p = 0.2016
Thickness (mm)				
≤ 1	7 (13.2)	74 (46.0)	1^b^	1^b^
1.01–2.00	7 (13.2)	39 (24.2)	2.07 (0.72–5.89)	1.55 (0.51–4.71)
2.01–4.00	28 (52.8)	38 (23.6)	5.85 (2.55–13.41)	2.42 (0.89–6.54)
> 4.00	11 (20.8)	10 (6.2)	7.45 (2.89–19.22)	3.13 (0.95–10.28)
χ^2^_1_ trend: p value			p < 0.0001	p = 0.0456
Ulceration				
Absent	29 (54.7)	113 (70.2)	1^b^	1^b^
Present	24 (45.3)	37 (23.0)	2.10 (1.22–3.61)	0.74 (0.40–1.39)
Unknown	–	11 (6.8)		
χ^2^_1_: p value			p = 0.0073	p = 0.3513
Regression				
Absent	37 (69.8)	95 (59.0)	1^2^	
Present	16 (30.2)	54 (33.5)	0.81 (0.45–1.45)	
Unknown	–	12 (7.5)		
χ^2^_1_: p value			p = 0.4687	
Mitoses (n/mm^2^)				
≤ 2	13 (24.5)	104 (64.6)	1^b^	1^b^
> 2	37 (69.8)	47 (29.2)	4.75 (2.52–8.94)	2.34 (1.11–4.97)
Unknown	3 (5.7)	10 (6.2)		
χ^2^_1_: p value			p < 0.0001	p = 0.0263
TIL^d^				
Absent	21 (39.6)	42 (26.1)	1^b^	
Brisk	9 (17.0)	32 (19.9)	0.62 (0.29–1.36)	
Non-Brisk	22 (41.5)	72 (44.7)	0.64 (0.35–1.16)	
Unknown	1 (1.9)	15 (9.3)		
χ^2^_1_ trend: p value			p = 0.1527	
Lymph nodes sentinel				
No suggest	15 (28.3)	81 (50.3)	1^b^	1^b^
Yes-negative	16 (30.2)	66 (41.0)	1.14 (0.56–2.31)	0.77 (0.35–1.69)
Yes-positive	22 (41.5)	14 (8.7)	5.24 (2.71–10.13)	2.60 (1.19–5.68)
χ^2^_1_ trend: p value			p < 0.0001	p = 0.0073

^a^Hazard ratio and 95% confidence interval (CI)

^b^Reference category

^c^Model including all significant terms in the univariate analysis

^d^Tumor infiltrating lymphocytes

By contrast, the risk of progression was not significantly associated with NM histotype (Table 2).

**Fig. 1:**
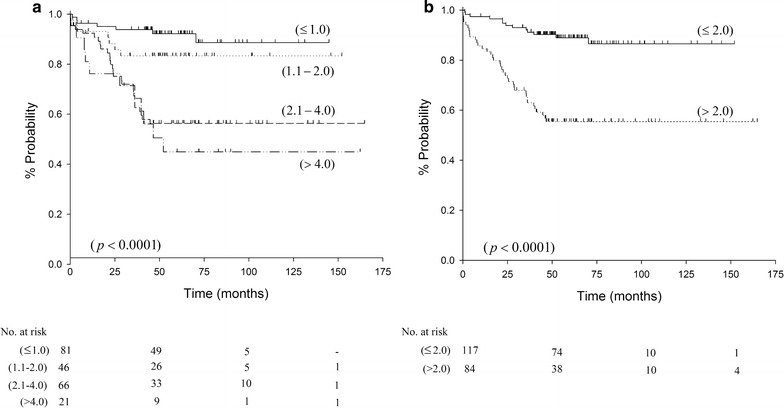
Probability of recurrence according to thickness (mm) (**a**) and mitotic rate (n./mm^2^) (**b**) in 214 cutaneous melanoma patients

The probability of recurrence according to BT and MR are reported in Fig. 1; at 5 years, the percentage of patients recurrence free were 45% (95% CI 23–65), 56% (95% CI 43–67), 83% (95% CI 68–92), and 92% (95% CI 83–96) for patients with BT > 4.00 mm, 2.01–4.00 mm, 1.01–2.00 mm, and ≤ 1 mm, respectively. In addition, the percentage of patients recurrence free were 55% (95% CI 44–65), and 89% (95% CI 81–94), for MR > 2, and ≤ 2 mitoses/mm^2^, respectively.

## Discussion

The NM subtype accounts for a large amount of thick melanomas, representing from 40 to 65% of all > 2 mm-thick melanomas [6, 11, 12]. In line with previous reports [6, 11–13], present results indicate that the NM subtype is significantly associated with higher BT and MR values at multivariate analysis. Furthermore, our results support previous observations that NM presents with higher MR values than SSM; indeed, the median number of mitoses reported for NM vs SSM was 2.5 and 1/mm^2^ respectively [13]. Different features of the two melanoma subtypes may derive from distinct genetic pathways and different origin [7, 14]. One could speculate that the hypothesized origin of NM from dermal stem cells and SSM from epidermal stem cells [14] could explain behavioral differences between the two melanoma subtypes. In particular, the growth of NM much faster than that of SSM should be considered as a feature closely associated with higher BT and MR scores among NM lesions [3].

By multivariate analysis, ulceration and regression were not significantly more frequent in NM as compared with SSM. In agreement with our results, Warycha et al. [13] did not find any significant difference between NM and SSM regarding the presence of regression. In contrast with our findings, this study however reported a significantly higher probability for NM to be found ulcerated [13].

No statistically significant difference was found in NM vs SSM in relation to tumor infiltrating lymphocytes (TILs) in the univariate analysis. This result is in contrast with that of other studies showing that patients with TILs more likely had nodular histology [15].

A limitation to our retrospective study was the selection bias and institutional variation in pathology assessment that could have potentially influenced our results.

NM was more frequently diagnosed in older men (≥ 50 years), particularly on the lower limbs or head and neck regions [6]. Consistently with Warycha et al. [13] study, we did not find any differences between NM and SSM regarding sex, age, and anatomical site (data not shown).

In agreement with prior reports [16–18], increasing BT, MR with > 2 mitoses/mm^2^, and the positivity of SNLB were significantly associated with an increased risk of recurrence in all melanoma cases (regardless of the subtype, NM or SSM). However, MR can be associated with SNLB positivity as reported by Mandalà et al. [19], whose study found that MR > 1 mitoses/mm^2^ in primary cutaneous melanoma with BT ≤ 1 mm was significantly predictive of metastasis in corresponding sentinel lymph node. The greater probability of recurrence with increasing BT from T1 to T4 and MR > 2 mitoses/mm^2^ is reported in Fig. 1.

In our study, NM was associated with a significant higher risk of recurrence in the univariate analysis but the association became not significant in the multivariate analysis. By contrast, Faut et al. [20] found that SLNB-negative patients with NM had a significant higher recurrence rate in the multivariate analysis (HR 1.82, p = 0.028). However, in our study, the HR was similar (1.62) but with a higher CI, resulting in a not significant p value. In addition, in Faut et al. [20] study, the significant association between NM and higher risk of recurrence was found solely in SLNB-negative and not in SLNB-positive patients. The differences between the two studies could depend on the different sample size of both NMs and SSMs.

O’ Connel et al. [21] have also reported a significant association between NM subtype and melanoma recurrence in patients with negative SLNB. However, in this latter study only SLNB- negative patients were included, while our series comprised both SLNB negative and positive patients [21].

In addition, and consistently with our results, other Authors found that NM was not significantly associated with higher risk recurrence in the multivariate analysis [22]. Although SSM has been found to have a better prognosis than NM, after adjusting for BT, a significant difference between two subtypes was not found [8]. Poor prognosis of NM could depend on a greater BT, as a consequence of a delayed diagnosis and not due to an intrinsic malignant effect of NM.

In our study, a significant association between ulceration and increased risk of recurrence was observed only in the univariate but not in the multivariate analysis, in agreement with previous studies that did not show an independent significant effect of ulceration on prognosis [17].

In addition, we did not find a statistically significant correlation between regression and recurrence in the univariate analysis. According to Tas et al. [23], the presence of histological regression plays no significant prognostic role in melanoma patients. However, the meta-analysis of 10 studies proposed that the regression can be considered a protective factor, probably depending on early activation of the host immune system against melanoma [24]. The prognostic value of histologic regression in melanoma remains controversial possibly due to lack of a standardized definition and objective criteria for histopathological classification of regression-associated parameters.

In our study, TIL response was not significantly associated with recurrence in the univariate analysis. In previous studies, TIL response was reported as an important independent prognostic indicator and the absence of TIL was considered as an independent predictor of SNL metastasis in melanoma [25].

We cannot exclude that the limited sample size, the participation of numerous centers in absence of lack of central histopathology review, as well as the relative short follow-up may account for this negative result. In multicentric studies, improved standardization in density and distribution of TILs is essential before the biologic and prognostic significance of histologic parameters related to immunity such as regression and TILs can be recognized.

Unfortunately, the limited series of cases in our study did not allow to rule out that NM could represent an independent prognostic factor.

## Conclusions

In summary, we herein showed that NM subtype was significantly associated with higher BT and MR scores. The NM subtype per se was not a prognostic factor, since it did not significantly affect the melanoma recurrence rate; the risk of melanoma recurrence was instead increased by higher BT, MR > 2/mm^2^, and SNLB positivity. Our study limitations did not allow drawing firm conclusions on the prognostic role of the NM subtype.

## Abbreviations

NM: nodular melanoma
SSM: superficial spreading melanoma
BT: breslow thickness
MR: mitotic rate
SNLB: lymph node sentinel biopsy
LMM: lentigo maligna melanoma
TIL: tumour infiltrating lymphocytes
