# Correction to: Clinicopathological predictors of recurrence in nodular and superficial spreading cutaneous melanoma: a multivariate analysis of 214 cases

**DOI:** 10.1186/s12967-018-1408-8

**Published:** 2018-02-20

**Authors:** Maria A. Pizzichetta, Daniela Massi, Mario Mandalà, Paola Queirolo, Ignazio Stanganelli, Vincenzo De Giorgi, Giovanni Ghigliotti, Stefano Cavicchini, Pietro Quaglino, Maria T. Corradin, Pietro Rubegni, Mauro Alaibac, Stefano Astorino, Fabrizio Ayala, Serena Magi, Laura Mazzoni, Maria Ausilia Manganoni, Renato Talamini, Diego Serraino, Giuseppe Palmieri, on behalf of the Italian Melanoma Intergroup (IMI)

**Affiliations:** 1 Division of Oncology B, CRO Aviano National Cancer Institute, Via Franco Gallini 2, 33081 Aviano, Italy; 2 Division of Pathological Anatomy, Department of Surgery and Translational Medicine, University of Florence, Florence, Italy; 3 Unit of Medical Oncology, Papa Giovanni XXIII Hospital, Bergamo, Italy; 4 Department of Medical Oncology, National Institute for Cancer Research, IRCCS San Martino, Genoa, Italy; 5 Skin Cancer Unit, Istituto Tumori Romagna (IRST), Meldola, Italy; 6 Department of Dermatology, University of Florence, Florence, Italy; 7 Clinic of Dermatology, IRCCS San Martino-IST, Genoa, Italy; 8 Department of Dermatology, Fondazione Ospedale Maggiore Policlinico IRCCS, Milan, Italy; 9 Dermatologic Clinic, Dept Medical Sciences, University of Torino, Turin, Italy; 10 Division of Dermatology, Pordenone Hospital, Pordenone, Italy; 11 Department of Dermatology, University of Siena, Siena, Italy; 12 Department of Dermatology, University of Padova, Padua, Italy; 13 Division of Dermatology, Celio Hospital, Rome, Italy; 14 National Cancer Institute, “Fondazione G. Pascale”-IRCCS, Naples, Italy; 15 Department of Dermatology, ASST degli Spedali Civili di Brescia, Brescia, Italy; 16 Unit of Epidemiology and Biostatistics, CRO Aviano National Cancer Institute, Aviano, Italy; 17 Unit of Cancer Genetics, Institute of Biomolecular Chemistry (ICB), National Research Council (CNR), Sassari, Italy; 18 Department of Dermatology, University of Parma, Parma, Italy

## Correction to: J Transl Med (2017) 15:227 10.1186/s12967-017-1332-3

In the original version of this article [1], published on 7 November 2017, affiliation 18 has been incorrectly assigned to the authors Serena Magi and Laura Mazzoni. They are only affiliated to the Skin Cancer Unit, Istituto Tumori Romagna (IRST), Meldola, Italy (affiliation 5).
